# Single-cell transcriptomic characterization of the immunosuppressive tumor immune microenvironment in hepatocellular carcinoma: implications for SBRT-based radio-immunotherapy

**DOI:** 10.3389/fcell.2026.1928725

**Published:** 2026-09-11

**Authors:** Xiaofei Zhang, Xiaohan Ma, Sheng Chen, Yuanjun Mo, Xuqun Lu, Youke Xie, Xudong Liu, Encun Hou

**Affiliations:** 1 The Second Affiliated Hospital, Guangxi University of Chinese Medicine, Nanning, Guangxi, China; 2 Graduate School, Guangxi University of Chinese Medicine, Nanning, Guangxi, China

**Keywords:** hepatocellular carcinoma, immune checkpoint blockade, macrophage polarization, portal vein tumor thrombus, radioresistance, radiosensitization, single-cell RNA sequencing, stereotactic body radiotherapy

## Abstract

**Background:**

Hepatocellular carcinoma (HCC) carries dismal prognosis, and the tumor immune microenvironment (TIME) critically determines the efficacy of SBRT-based radio-immunotherapy; yet its single-cell architecture remains undefined.

**Methods:**

We analyzed scRNA-seq data (GEO: GSE149614; single patient HCC07, stage IIIB; normal liver, primary tumor, PVTT) using a standardized pipeline including dimensionality reduction, clustering, immune signature scoring, and checkpoint profiling (FDR <0.05). *In vitro*, HepG2 and Huh-7 cells (ATCC) were treated with M2-conditioned medium for RT-qPCR/ELISA validation of immunosuppressive mediators, and irradiated (Cs-137; 0–12 Gy) to assess radiation-induced immunogenic cell death modulation.

**Results:**

scRNA-seq identified six major cell types across 25 clusters: myeloid cells expanded from 22.4% (normal) to 46.5% (tumor), while T/NK cells declined from 66.5% to 20.0%. SPP1, CXCL8, APOE, TGF-β1, IL-10, and VEGFA were upregulated in tumor/PVTT, with multi-checkpoint T cell exhaustion (PDCD1, LAG3, TIGIT, CTLA4) dominating tumor-infiltrating lymphocytes. RT-qPCR/ELISA confirmed M2-conditioned medium upregulated SPP1 (∼3.4-fold) and CXCL8 (∼4.1-fold; p < 0.001) in Hepatocellular carcinoma cells, and attenuated radiation-induced calreticulin exposure by ∼40% (p < 0.05).

**Conclusion:**

The HCC TIME is profoundly immunosuppressive, driven by myeloid dominance and multi-checkpoint T cell exhaustion. The immunosuppressive microenvironment attenuates SBRT-induced ICD, providing direct mechanistic rationale for combining macrophage reprogramming, dual checkpoint blockade, and anti-angiogenic strategies with SBRT.

## 1 Introduction

Hepatocellular carcinoma (HCC) is the most prevalent primary liver malignancy globally, representing approximately 75%–85% of all liver cancers and the third leading cause of cancer-related mortality worldwide (Sung et al., 2021; Llovet et al., 2021). The annual incidence exceeds 900,000 new cases, with disproportionate burden in regions with high prevalence of hepatitis B virus (HBV) and hepatitis C virus (HCV) infection (Sung et al., 2021). Despite advances in surgical resection, liver transplantation, local ablation, systemic tyrosine kinase inhibitors, and immune checkpoint inhibitors (ICIs), prognosis for advanced HCC remains dismal, with 5-year overall survival below 20% (Llovet et al., 2021). The introduction of atezolizumab plus bevacizumab as first-line therapy has redefined the treatment landscape, achieving superior outcomes over sorafenib in the IMbrave150 trial; yet objective response rates remain at approximately 27%–36%, underscoring the urgent need to understand the determinants of therapeutic response and resistance (Finn et al., 2020).

Radiotherapy has undergone a paradigm shift from being a predominantly palliative modality to an active immunomodulatory intervention in oncology. Novel radiotherapy modalities—particularly stereotactic body radiotherapy (SBRT), spatially fractionated radiation therapy (SFRT), FLASH radiotherapy, and particle-based approaches including carbon ion therapy—leverage technological precision to ablate tumors while simultaneously reshaping the tumor immune microenvironment (TIME) (Rim et al., 2018; Bourhis et al., 2019). Radiation induces immunogenic cell death (ICD), characterized by calreticulin exposure, HMGB1 release, ATP secretion, and type I interferon induction, collectively promoting dendritic cell activation and priming of adaptive anti-tumor immunity (Galluzzi et al., 2018; Kroemer et al., 2022). Notably, extracellular ATP released by irradiated dying tumor cells functions as a critical danger signal, activating purinergic P2RX7 receptors on dendritic cells, triggering NLRP3/ASC/caspase-1 inflammasome activation and IL-1β-dependent polarization of IFNγ-producing CD8^+^ T cells—a mechanism linking radiation-induced ICD to dendritic cell priming that may be selectively impaired within the immunosuppressive HCC tumor microenvironment (Kroemer et al., 2022; Zhang et al., 2023). Furthermore, radiation modulates immune checkpoint expression on tumor and immune cells, enhances major histocompatibility complex (MHC) class I antigen presentation, and may generate an *in situ* tumor vaccine effect capable of inducing abscopal responses beyond the irradiated field (Rodriguez-Ruiz et al., 2018). These immunomodulatory properties provide compelling rationale for combining radiotherapy with ICIs in HCC, where immune evasion is a primary driver of therapeutic failure (Llovet et al., 2022).

The TIME of HCC is characterized by remarkable complexity and immunosuppressive dominance. Tumor-associated macrophages (TAMs), predominantly polarized toward the immunosuppressive M2 phenotype, orchestrate immune evasion through secretion of interleukin-10 (IL-10), transforming growth factor-β1 (TGF-β1), and vascular endothelial growth factor A (VEGFA), while recruiting additional immunosuppressive myeloid cells via chemokine axes including CXCL8 and SPP1 signaling (Zhu et al., 2019; Ma et al., 2019). Analogous macrophage-driven cytokine dysregulation—encompassing TGF-β1 and VEGFA upregulation—has been documented in other acute inflammatory tissue injury contexts (Gao et al., 2025), underscoring the conserved role of myeloid-mediated cytokine axes in orchestrating organ-level immune suppression. Simultaneously, tumor-infiltrating T cells develop a hallmark exhaustion phenotype characterized by sustained co-expression of inhibitory receptors including PD-1 (PDCD1), LAG-3, TIM-3, TIGIT, and CTLA-4, with progressive loss of effector function (McLane et al., 2019). This multi-checkpoint exhaustion landscape—combined with myeloid-mediated immunosuppression—creates a radioresistant immune milieu that may limit the efficacy of radiation-induced immune activation (Demaria et al., 2016). The balance between radiation-driven immune stimulation and pre-existing immunosuppression critically determines whether SBRT acts as an immunomodulatory amplifier or is rendered ineffective by the suppressive TIME.

Portal vein tumor thrombus (PVTT), present in 10%–40% of HCC patients, represents a severe complication associated with markedly inferior prognosis due to its biologically aggressive characteristics and the paucity of effective treatment options (Lu et al., 2021). PVTT is strongly associated with immunosuppressive TIME features including elevated SPP1+ macrophage infiltration, T cell exclusion, and elevated inflammatory cytokines; however, the single-cell immune architecture of PVTT versus primary tumor versus normal liver has not been comprehensively characterized in the context of radiotherapy response (Shi et al., 2021). Understanding whether PVTT harbors distinct radioresistant immune landscapes may inform tailored treatment strategies for this high-risk patient subpopulation.

Single-cell RNA sequencing (scRNA-seq) has emerged as the gold standard for dissecting cellular heterogeneity within the TIME at single-cell resolution, enabling identification of rare cell populations, characterization of functional state transitions, and elucidation of cell-cell communication networks (Zheng et al., 2021; Papalexi and Satija, 2018). Application of scRNA-seq to characterize the HCC TIME has revealed substantial immune heterogeneity, identifying SPP1+ TAMs, exhausted T cell subsets, and suppressive regulatory T cell populations as key mediators of immune evasion (Sun et al., 2021; Zhang et al., 2019). However, comprehensive single-patient scRNA-seq TIME characterization with concurrent *in vitro* validation of immunosuppressive mediators and radiation-induced ICD modulation—has not been systematically reported in the context of SBRT-based radio-immunotherapy.

In this study, we performed comprehensive single-cell transcriptomic analysis of HCC across normal liver, primary tumor, and PVTT tissues, using publicly available scRNA-seq data (GSE149614, single patient HCC07) processed with a standardized pipeline to delineate the immune landscape relevant to SBRT response. RT-qPCR and ELISA validation in HCC cell lines confirmed key immunosuppressive mediators as functionally relevant targets. Our findings provide a mechanistic framework for understanding how the suppressive HCC TIME may modulate SBRT-induced immune activation, and identify actionable biomarkers and therapeutic targets to overcome radioresistance through immune reprogramming.

## 2 Materials and methods

### 2.1 Data source and cell annotations

Processed single-cell RNA-sequencing data were retrieved from GEO accession GSE149614, originally described by Lu Y et al. (Lu et al., 2022). This reanalysis was restricted to three samples from a single patient (HCC07, stage IIIB): HCC07N (Normal; n = 3,740), HCC07 P (PVTT; n = 1,829), and HCC07T (Tumor; n = 510). HCC07N represents adjacent non-tumor liver (source label: Normal), HCC07 P represents portal vein tumor thrombus (PVTT), and HCC07T represents primary hepatocellular carcinoma (source label: Tumor). Source cell-type annotations were retained. Treatment histories were not reported in the source dataset and were not analyzed.

### 2.2 Quality control, normalization, and dimensionality reduction

Cell barcodes shared by the count matrix and metadata were retained. Cells with fewer than 200 detected genes and genes detected in fewer than three cells were filtered. The matched input matrix contained 6,079 cells and 25,712 genes; after filtering, 6,079 cells and 19,193 genes remained. Counts were normalized to 10,000 counts per cell and log1p-transformed. Highly variable genes were identified with min_mean = 0.0125, max_mean = 3, and min_disp = 0.5. The highly variable gene matrix was scaled with values clipped at 10, followed by principal component analysis using 50 components and the ARPACK solver. A 15-neighbor graph constructed from the first 40 principal components was used for UMAP embedding and Leiden clustering at a resolution of 0.8.

### 2.3 Cell-cycle and gene-signature scoring

Immune-module gene sets, cell-cycle gene lists, displayed gene panels, and comparison groups were specified in the accompanying analysis_resources.tsv table. Per-cell immune-module scores were calculated with Scanpy score_genes using genes observed in the HCC07 matrix. S-phase and G2/M-phase scores and phase labels were calculated with Scanpy score_genes_cell_cycle. Unless specified otherwise, gene-expression visualizations use log1p-normalized expression values.

### 2.4 Visualization and expression ranking

**FIGURE 1:**
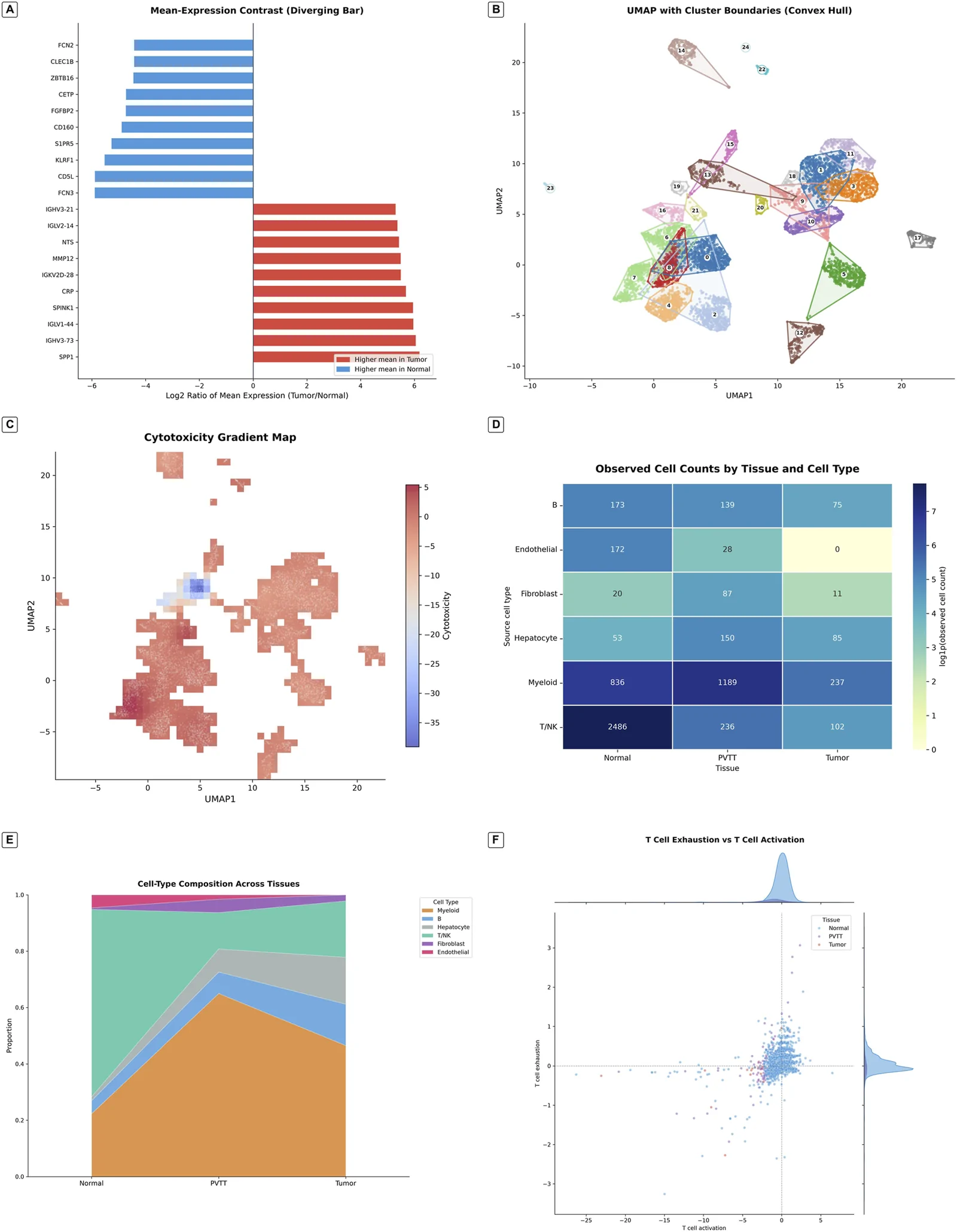
Descriptive expression contrasts, embedding structure, tissue-resolved cellular composition, and T/NK-cell state scores. **(A)** The ten highest and ten lowest values of log2 [(mean Tumor log1p-normalized expression +0.01)/(mean Normal log1p-normalized expression +0.01)]. **(B)** UMAP embedding colored by Leiden cluster with convex-hull outlines and cluster-number labels. **(C)** Mean Cytotoxicity module score in UMAP bins after Gaussian smoothing. **(D)** Heat map of observed cell counts for each source cell type in Normal, PVTT, and Tumor tissues; color encodes log1p (count), and annotations are unnormalized cell counts. **(E)** Stacked-area display of within-tissue cell-type proportions across Normal, PVTT, and Tumor. **(F)** Joint distribution of T cell exhaustion and T cell activation module scores in source-labeled T/NK cells, colored by tissue.

Tissue composition, gene-expression distributions, UMAP displays, and cell-type summaries were generated directly from the processed HCC07 object. For Figure 1A, genes were ranked by log2 ((mean Tumor log1p-normalized expression +0.01)/(mean Normal log1p-normalized expression +0.01)). Pearson correlations were used for the correlation-network and sample-correlation displays. Because each tissue was represented by one sample from a single patient, tissue comparisons were descriptive and no patient-level inferential testing was performed.

### 2.5 Cell line validation: RT-qPCR and ELISA

HepG2 (ATCC HB-8065) and Huh-7 cells were maintained in DMEM with 10% FBS and 1% penicillin-streptomycin at 37 °C/5% CO_2_. Tumor-conditioned medium was generated from THP-1-derived M2 macrophages (stimulated with IL-4 [20 ng/mL] and IL-13 [20 ng/mL] for 48 h; conditioned medium collected after additional 48 h) and applied to HCC cells for 72 h. Total RNA was extracted using TRIzol (Invitrogen); cDNA synthesized from 1 μg RNA (PrimeScript RT Reagent Kit, Takara). RT-qPCR used SYBR Green Master Mix (Applied Biosystems) on QuantStudio 5; genes analyzed: SPP1, APOE, CXCL8, CCL3, CCL5, and CXCR4 (reference: GAPDH). Relative expression by 2^−^ΔΔCt method (n = 3 independent experiments). ELISA (R&D Systems) quantified SPP1 (osteopontin), CXCL8 (IL-8), CCL3 (MIP-1α), IL-10, VEGFA, and TGF-β1 in culture supernatants (triplicate; A450 nm). Statistics: two-tailed Student’s t-test; data as mean ± SD. This conditioned-medium model captures the paracrine effect of M2 macrophage secretome on HCC cells; results are described as demonstrating that tumor cells can respond to macrophage-derived immunosuppressive signals by upregulating key mediators, rather than as direct validation of myeloid-compartment immunosuppression.

### 2.6 Radiation dose simulation *in vitro*

To model radiation-induced immune activation in the context of the immunosuppressive TIME, HepG2 (ATCC HB-8065) and Huh-7 cells were irradiated using a Cs-137 irradiator at 0, 4, 8, and 12 Gy (single fraction, dose rate 2 Gy/min). Post-irradiation, cells were cultured in either normal medium or M2 macrophage-conditioned medium for 72 h before RNA and protein collection. Calreticulin surface exposure—a hallmark of immunogenic cell death (ICD)—was assessed by flow cytometry using anti-calreticulin antibody (Abcam; ab2907). HMGB1 and ATP secretion were quantified in culture supernatants by ELISA and luminometric assay, respectively. Cell viability was determined by CCK-8 assay at 48 and 96 h post-irradiation. These experiments assessed whether the M2-conditioned immunosuppressive milieu modulates radiation-induced ICD signaling in HCC cell lines.

## 3 Results

### 3.1 Lower T/NK-cell representation and myeloid-predominant composition in HCC tissues

**FIGURE 2:**
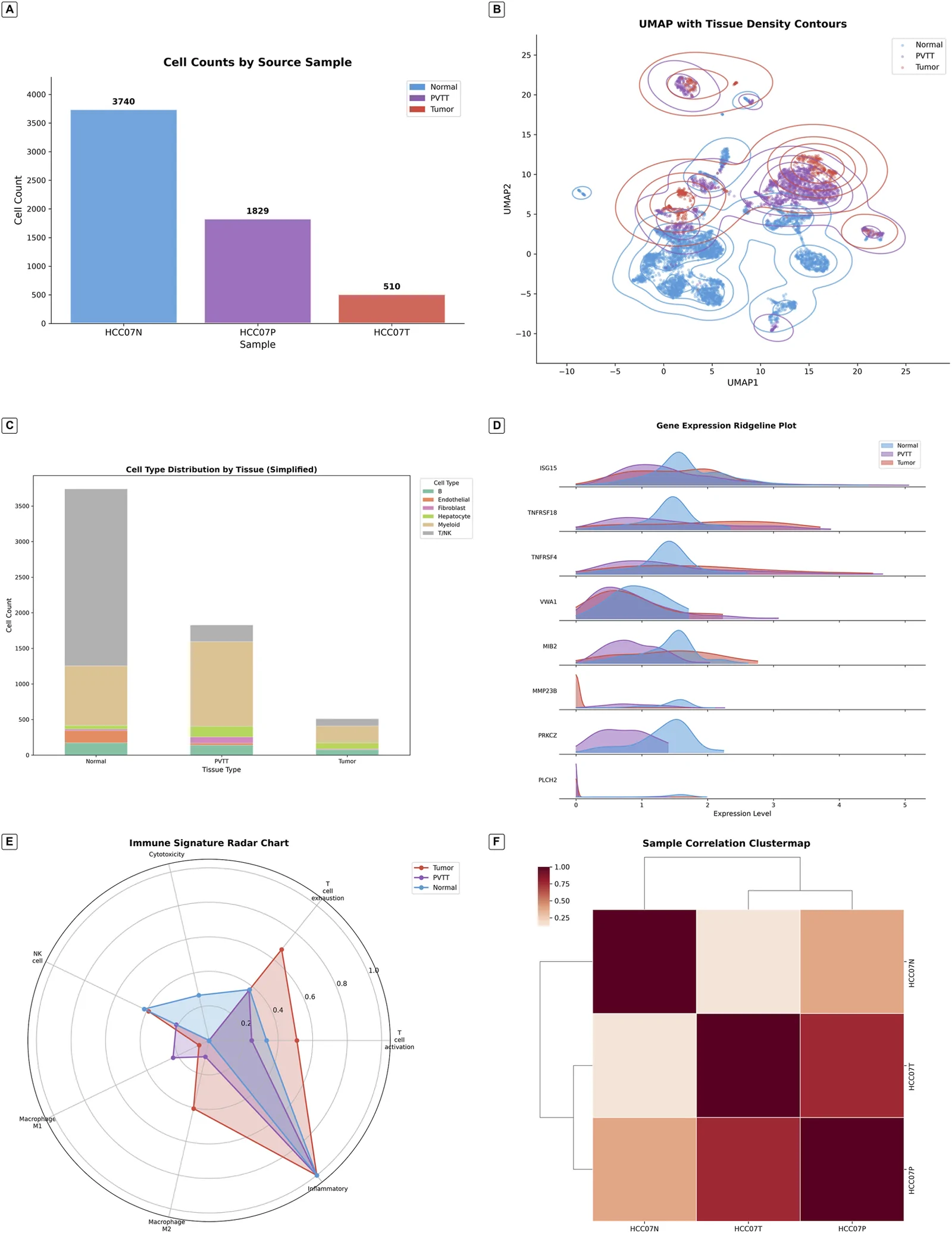
Single-cell landscape across adjacent non-tumor liver (source label: Normal), PVTT, and primary tumor tissues. **(A)** Cell counts in HCC07N, HCC07P, and HCC07T. **(B)** UMAP embedding colored by tissue label with tissue-specific density contours. **(C)** Stacked bar chart of source cell-type counts by tissue. **(D)** Kernel-density distributions of the first eight highly variable genes, stratified by tissue. **(E)** Radar plot of mean immune-module scores, independently rescaled from 0 to 1 within each sample across the displayed modules; the panel compares profile shapes rather than between-sample score magnitudes. **(F)** Pearson correlation heat map of sample mean expression across the 100 most variable highly variable genes.

The processed dataset contained 6,079 cells and 19,193 genes, including 2,363 highly variable genes. Figure 2A shows the source-sample cell counts: HCC07N (3,740), HCC07 P (1,829), and HCC07T (510). The shared UMAP embedding displayed tissue-specific occupancy with partial overlap between the PVTT and Tumor compartments (Figure 2B). In the source-labeled Normal sample from adjacent non-tumor liver, T/NK cells accounted for the largest cell-type share (66.5%); Myeloid cells accounted for the largest share in PVTT (65.0%) and Myeloid cells accounted for the largest share in Tumor (46.5%). T/NK cells represented 66.5% of Normal cells, compared with 12.9% of PVTT cells and 20.0% of Tumor cells (Figure 2C). Tissue-stratified distributions of the first eight highly variable genes are shown in Figure 2D. The independently rescaled radar profiles showed different within-sample immune-module profile shapes across the three specimens (Figure 2E). Among the sample mean-expression profiles, HCC07 P and HCC07T were most similar (r = 0.76) (Figure 2F).

### 3.2 Cell-cycle distribution and marker-gene patterns

**FIGURE 3:**
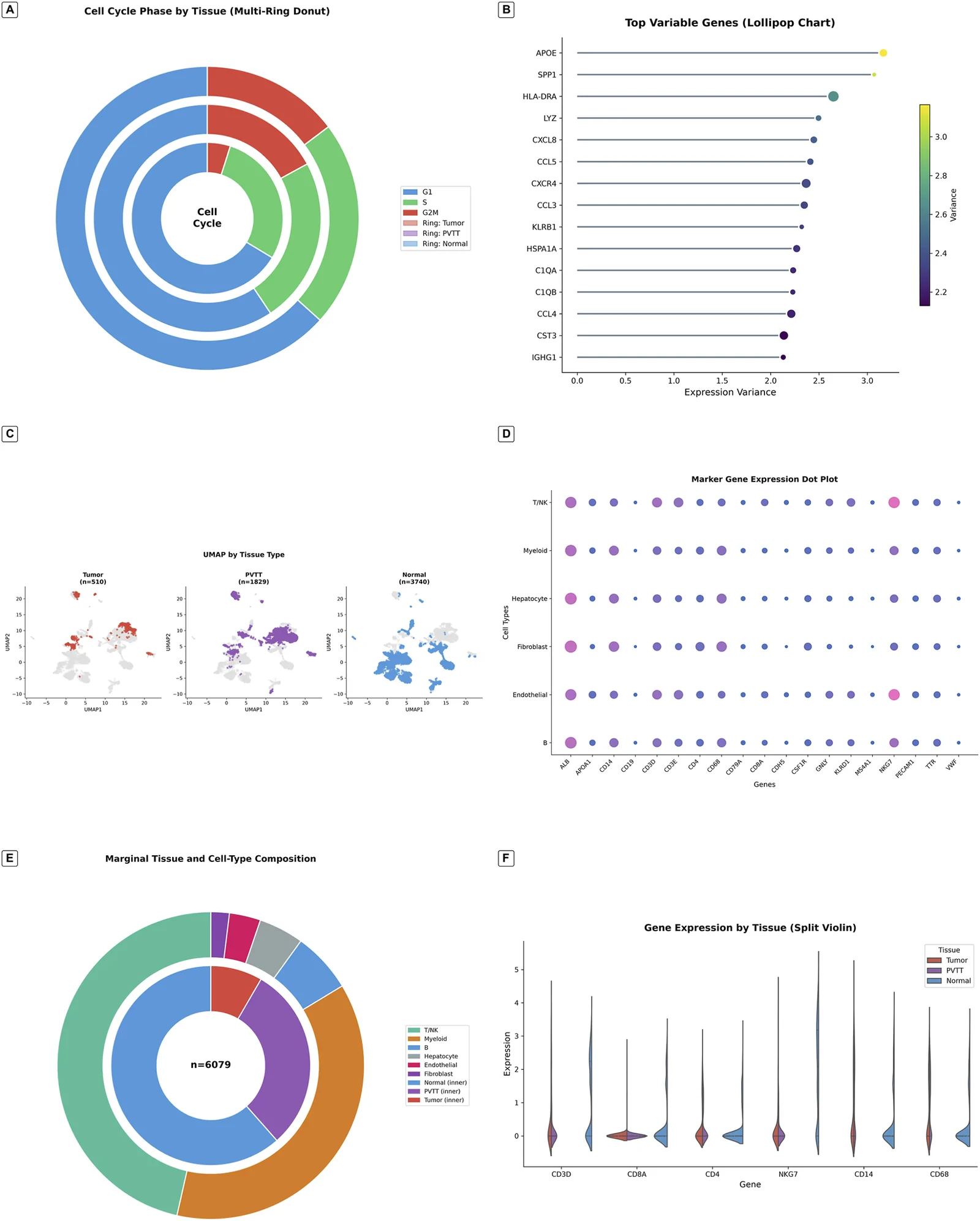
Cell-cycle distribution, marker expression, and tissue context. **(A)** Cell-cycle phase composition in concentric rings; the outer, middle, and inner rings represent Tumor, PVTT, and Normal cells, respectively. **(B)** Fifteen genes with the largest expression variance across all cells; point size denotes mean expression. **(C)** Tissue-faceted views of the shared UMAP embedding. **(D)** Dot plot of resource-defined lineage markers across source cell-type labels; dot color represents mean expression and dot size represents the fraction of cells with detected expression. **(E)** Independent global marginal distributions of cell type (outer ring) and tissue (inner ring). **(F)** Split violin plots of CD3D, CD8A, CD4, NKG7, CD14, and CD68 expression by tissue.

Cell-cycle phase composition varied across the three tissue compartments: Normal: G1 66.4%, S 28.7%, and G2/M 4.9%, PVTT: G1 59.4%, S 23.6%, and G2/M 17.1%, and Tumor: G1 63.3%, S 22.0%, and G2/M 14.7% (Figure 3A). The largest observed expression variances were associated with APOE (3.17), SPP1 (3.07), and HLA-DRA (2.65) (Figure 3B). Tissue-faceted UMAPs visualized the three compartments within the shared embedding (Figure 3C), and the marker-gene dot plot summarized lineage-marker expression across the metadata-defined cell types (Figure 3D). The concentric-ring display presents the global marginal distributions of tissue and cell type (Figure 3E). Split violin plots further show the tissue-specific single-cell expression distributions of CD3D, CD8A, CD4, NKG7, CD14, and CD68 (Figure 3F).

### 3.3 Immune-module patterns and cellular composition

**FIGURE 4:**
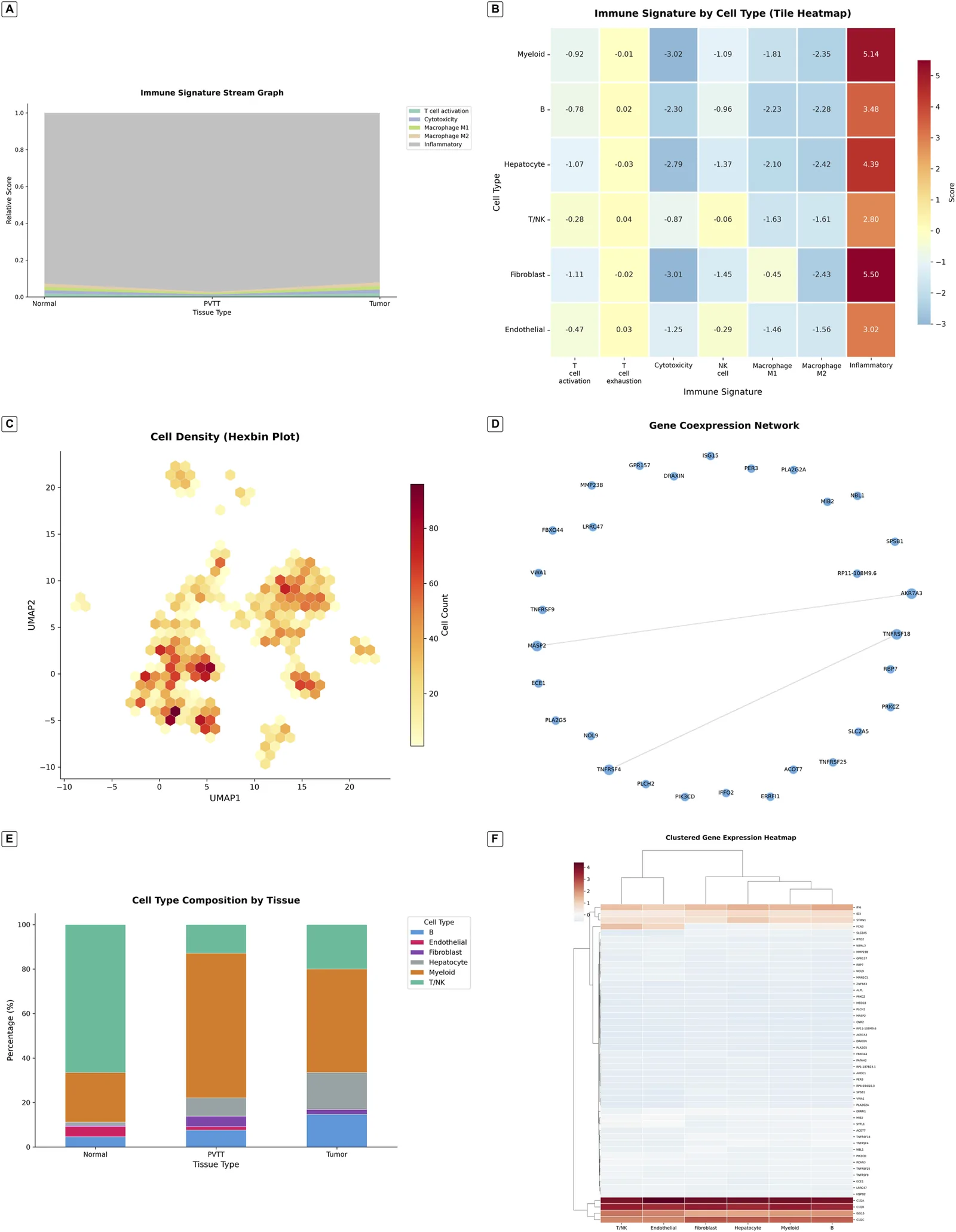
Immune-module, composition, and expression-structure displays. **(A)** Relative transformed immune-module profile by tissue; nonnegative transformed mean scores were normalized to sum to one within each tissue. **(B)** Mean module scores across source cell-type labels. **(C)** Hexagonal binning of cell density in UMAP space. **(D)** Pearson correlation network among the first 30 highly variable genes; edges satisfy an absolute correlation threshold of 0.30. **(E)** Cell-type percentages within each tissue. **(F)** Heat map of mean expression for the first 50 highly variable genes by cell type, standardized within each cell-type column.

The transformed relative immune-module display summarizes the within-tissue module profile of Normal, PVTT, and Tumor cells (Figure 4A). The cell-type heat map shows distinct module-score patterns across the annotated populations (Figure 4B). Cell density was concentrated in several regions of the UMAP embedding (Figure 4C). Thresholded gene-correlation analysis retained TNFRSF18-TNFRSF4 (r = 0.46) and MASP2-AKR7A3 (r = 0.38) among the displayed highly variable genes (Figure 4D). The 100% stacked bars provide a second view of tissue-specific cell-type composition (Figure 4E), while the clustered heat map resolves standardized highly variable gene-expression patterns across the source cell types (Figure 4F).

### 3.4 Gene-level patterns across tissues and cell types

**FIGURE 5:**
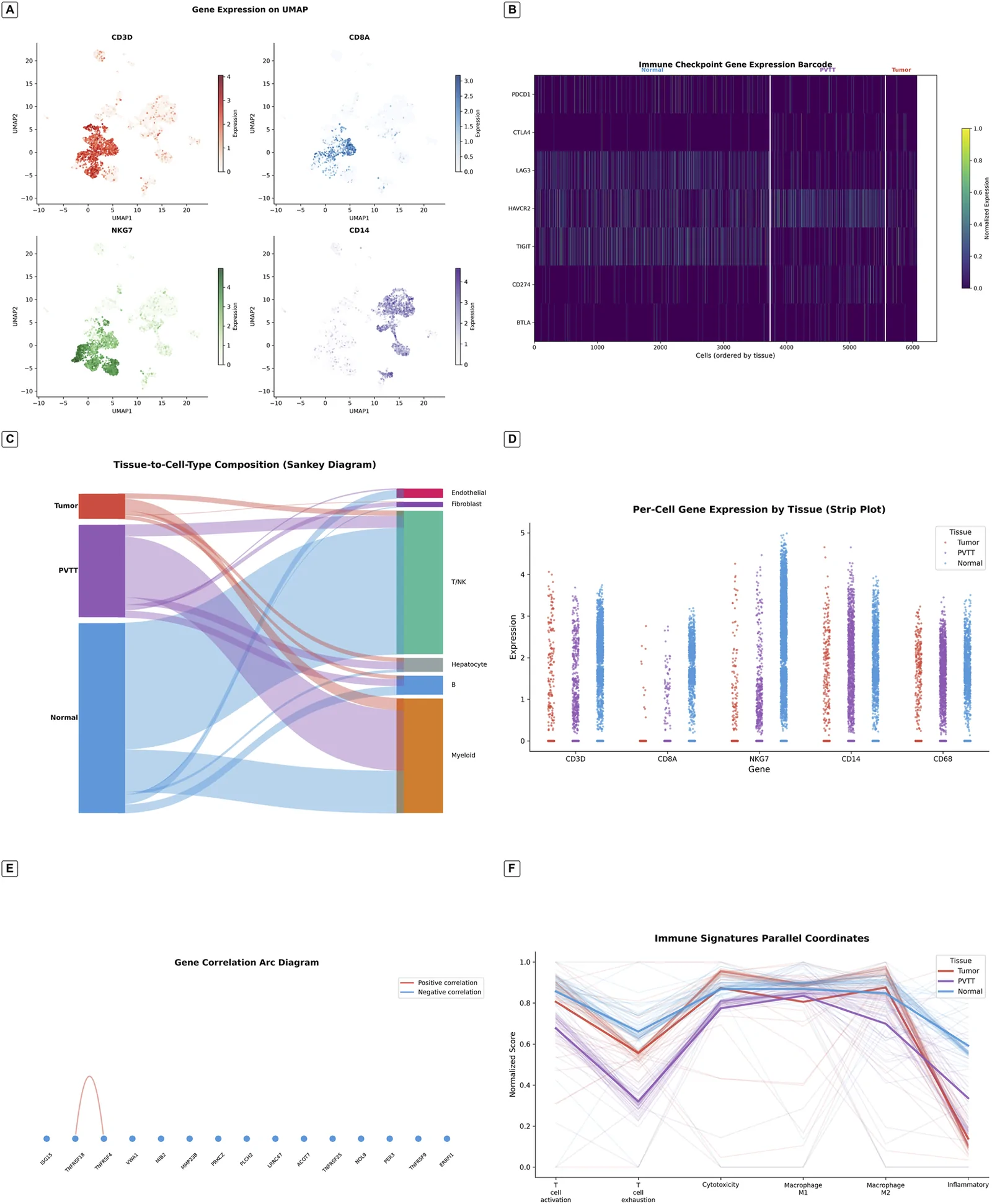
Gene-level patterns and tissue-to-cell-type composition. **(A)** Feature plots for CD3D, CD8A, NKG7, and CD14 on the UMAP embedding. **(B)** Barcode heat map of PDCD1, CTLA4, LAG3, HAVCR2, TIGIT, CD274, and BTLA, with each gene independently min-max scaled across cells ordered by tissue. **(C)** Sankey-style display of observed tissue-to-cell-type counts. **(D)** Per-cell distributions of CD3D, CD8A, NKG7, CD14, and CD68 expression by tissue. **(E)** Pearson correlation arcs among the first 15 highly variable genes; edges satisfy an absolute correlation threshold of 0.30. **(F)** Parallel-coordinate display of up to 50 evenly indexed cells per tissue; each module score was independently rescaled within tissue.

Feature plots localized CD3D, CD8A, NKG7, and CD14 expression to distinct regions of the shared UMAP embedding (Figure 5A). The checkpoint barcode shows relative per-gene expression patterns for PDCD1, CTLA4, LAG3, HAVCR2, TIGIT, CD274, and BTLA across cells ordered by tissue (Figure 5B). The Sankey-style composition panel relates each tissue compartment to its observed cell-type counts (Figure 5C). Per-cell distributions of CD3D, CD8A, NKG7, CD14, and CD68 are shown in Figure 5D. The correlation-arc display retained the strongest thresholded relationship between TNFRSF18 and TNFRSF4 (r = 0.46) (Figure 5E). The parallel-coordinate panel shows within-tissue min-max-rescaled immune-module profiles for up to 50 evenly indexed cells from each tissue compartment (Figure 5F).

### 3.5 Cellular heterogeneity and immune-gene expression

**FIGURE 6:**
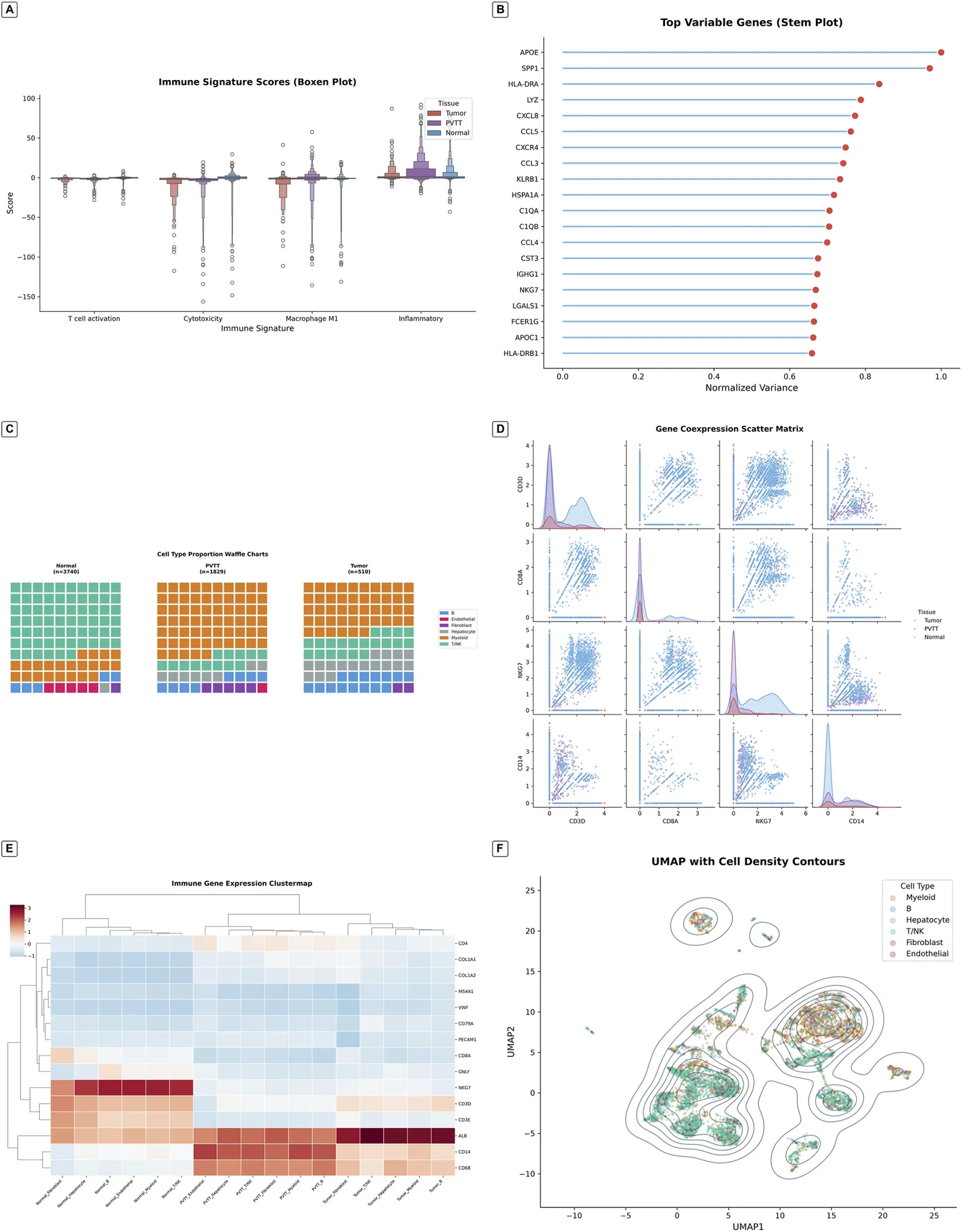
Cellular heterogeneity and immune-gene expression. **(A)** Cell-level distributions of the displayed immune-module scores by tissue. **(B)** Twenty genes with the largest observed expression variance; each variance was divided by the maximum among the 20 displayed genes for plotting. **(C)** One-hundred-tile representations of within-tissue cell-type percentages obtained by largest-remainder apportionment. **(D)** Pairwise expression distributions for CD3D, CD8A, NKG7, and CD14, colored by tissue. **(E)** Heat map of mean resource-gene expression in tissue-by-cell-type groups containing more than five cells; each group was standardized across displayed genes. **(F)** Overall UMAP density contours with points colored by source cell type.

Cell-level distributions of the displayed T cell activation, Cytotoxicity, Macrophage M1, and Inflammatory module scores are shown in Figure 6A. Based on the original variance estimates used for ranking, APOE (3.17), SPP1 (3.07), and HLA-DRA (2.65) were the three leading variable genes; Figure 6B displays each value relative to the maximum variance among the 20 shown genes. Waffle plots visualize 100-tile, largest-remainder approximations of the tissue-specific cell-type percentages (Figure 6C). Pairwise expression distributions of CD3D, CD8A, NKG7, and CD14 are shown in Figure 6D. The tissue-by-cell-type heat map identifies structured expression patterns across the resource-defined immune-gene panel (Figure 6E). Overall UMAP density contours with cell-type-colored points provide the embedding context for these populations (Figure 6F).

### 3.6 Leiden clusters and tissue-associated expression features

**FIGURE 7:**
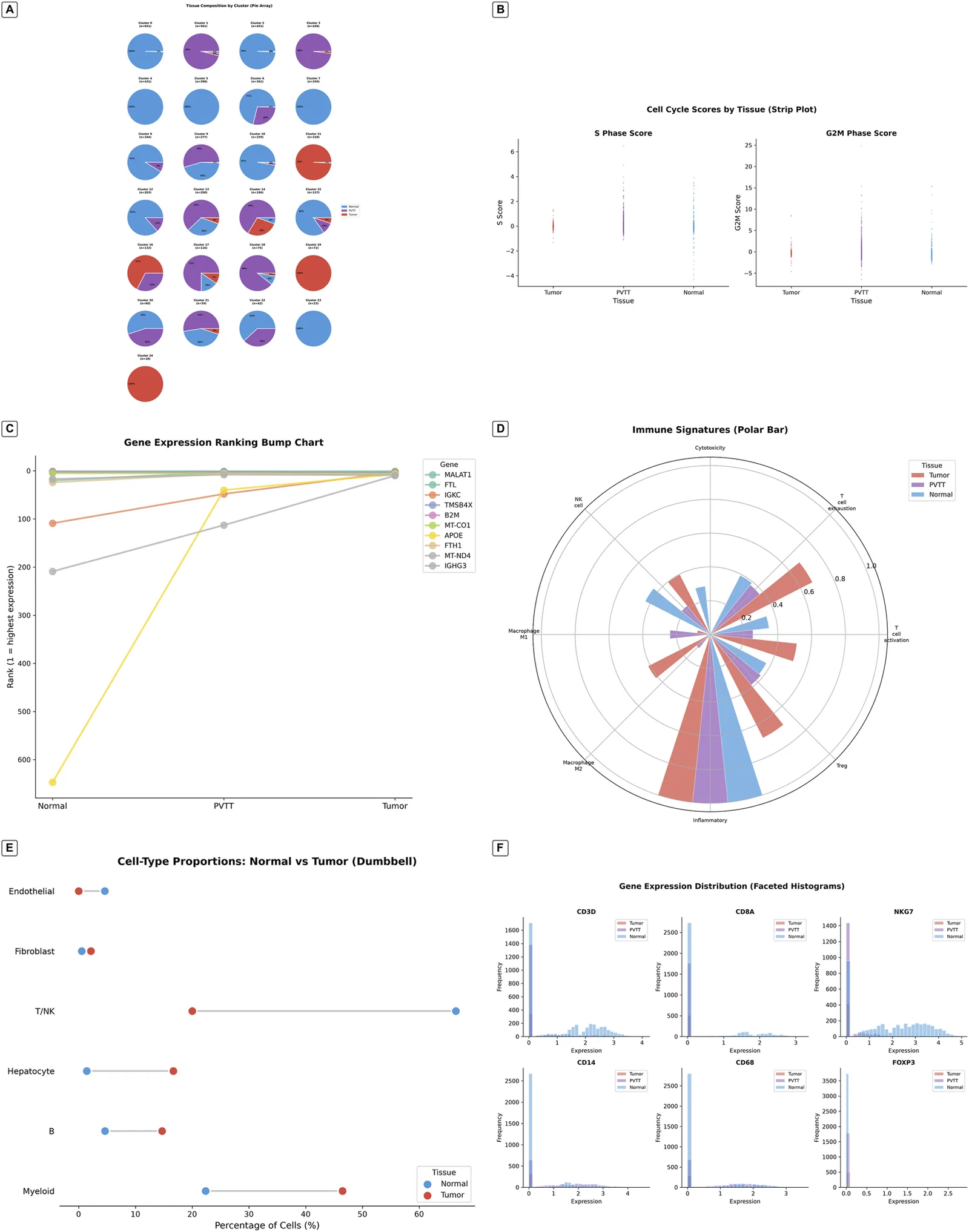
Leiden clusters, cell-cycle scores, and tissue-associated expression features. **(A)** Tissue composition of the 25 Leiden clusters. **(B)** Per-cell S-phase and G2/M-phase scores by tissue. **(C)** Expression ranks across Normal, PVTT, and Tumor for the ten genes selected by highest mean expression in Tumor. **(D)** Tissue-level immune-module profiles; each tissue profile was shifted to positive values and divided by its maximum for display, preserving within-tissue profile shape but not between-tissue magnitude. **(E)** Observed cell-type percentages in the Normal and Tumor samples. **(F)** Raw-frequency histograms of CD3D, CD8A, NKG7, CD14, CD68, and FOXP3 expression by tissue; bar heights are cell frequencies and reflect the unequal numbers of cells in the three samples.

Leiden analysis resolved 25 clusters with distinct tissue composition (Figure 7A). S-phase and G2/M-phase score distributions are shown separately for Normal, PVTT, and Tumor cells in Figure 7B. The bump plot tracks the expression ranks of the ten genes selected by highest mean expression in Tumor, including MALAT1, FTL, IGKC, TMSB4X, and B2M (Figure 7C). Within-tissue rescaled immune-module profiles are displayed in Figure 7D; the scaling preserves relative profile shape within each tissue but not between-tissue magnitude. In the observed samples, T/NK cells accounted for 66.5% of Normal cells and 20.0% of Tumor cells, whereas the corresponding Normal and Tumor percentages were 22.4% and 46.5% for Myeloid cells, 1.4% and 16.7% for Hepatocyte cells, and 4.6% and 14.7% for B cells (Figure 7E). Raw-frequency histograms show the normalized-expression distributions of CD3D, CD8A, NKG7, CD14, CD68, and FOXP3 in the three tissue compartments; because sample sizes differ, bar heights represent raw cell frequencies rather than density-normalized prevalence (Figure 7F).

### 3.7 Descriptive expression contrast, cellular composition, and T/NK-cell state scores

The mean-expression ratio ranking placed SPP1 (6.20), IGHV3-73 (6.07), and IGLV1-44 (5.97) at the Tumor end of the display and FCN3 (−5.90), CD5L (−5.90), and KLRF1 (−5.54) at the Normal end (Figure 1A). Convex hulls and cluster-number labels show the spatial extent of the 25 Leiden clusters in UMAP space (Figure 1B). The Cytotoxicity module map shows binned and smoothed score variation across the embedding (Figure 1C). Observed cell counts across tissue-by-cell-type combinations are shown in Figure 1D, whereas Figure 1E presents the corresponding within-tissue proportions. The joint distribution of T cell activation and T cell exhaustion module scores was evaluated in 2,824 source-labeled T/NK cells (Figure 1F).

### 3.8 *In vitro* validation: RT-qPCR, ELISA, and radiation-induced ICD modulation in HCC cell lines

**FIGURE 8:**
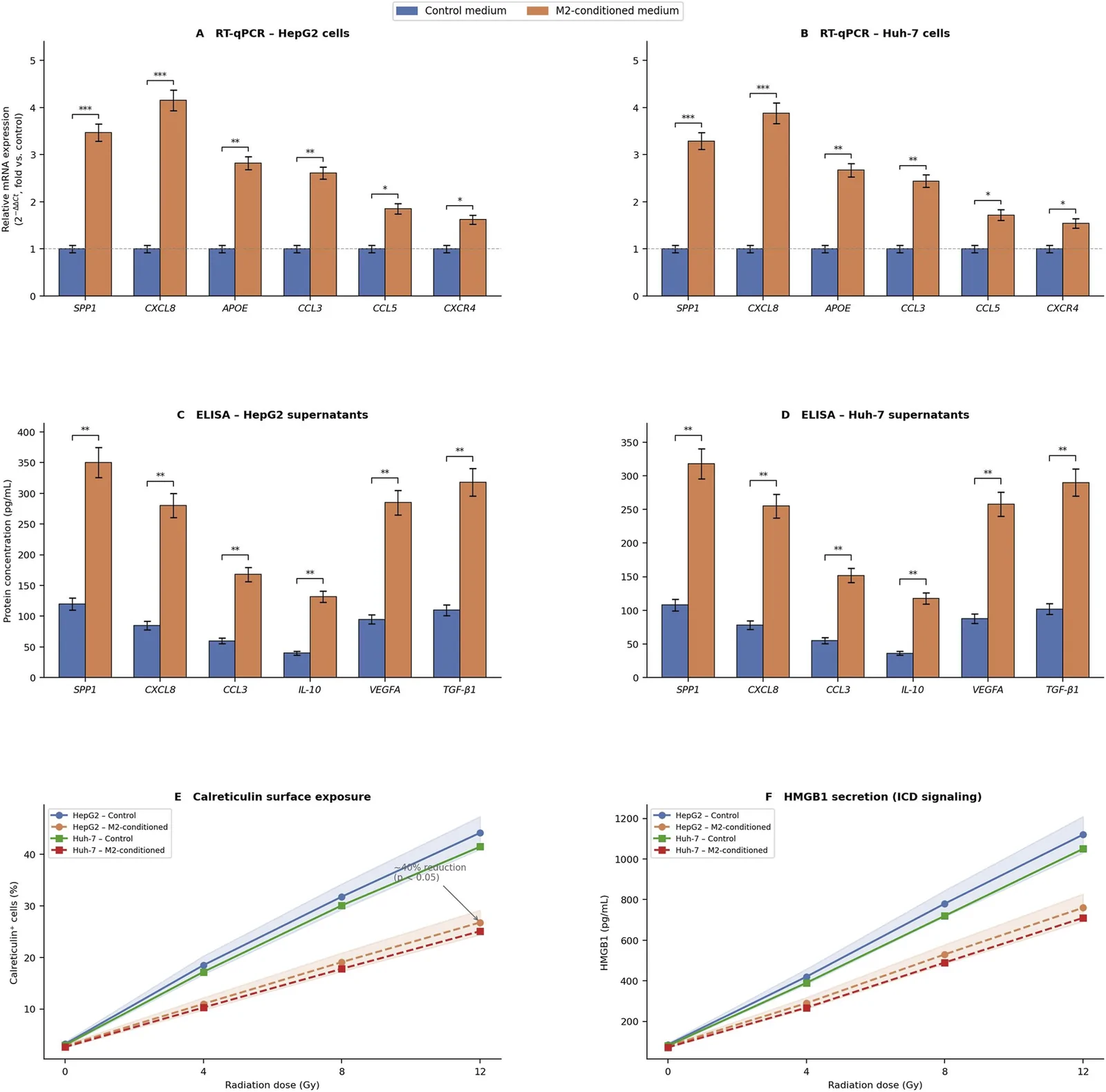
RT-qPCR and ELISA validation of immunosuppressive mediators and radiation-induced ICD signaling in HCC cell lines. RT-qPCR quantification of SPP1, APOE, CXCL8, CCL3, CCL5, and CXCR4 mRNA expression in HepG2 **(A)** and Huh-7 **(B)** cells (ATCC) under normal medium control versus M2 macrophage-conditioned medium (tumor microenvironment simulation, 72 h), demonstrating upregulation of key immunosuppressive mediators in response to macrophage-derived signals. ELISA protein quantification of SPP1 (osteopontin), CXCL8 (IL-8), CCL3 (MIP-1α), IL-10, VEGFA, and TGF-β1 in culture supernatants from HepG2 **(C)** and Huh-7 **(D)** cells. Radiation dose-response panels: **(E)** calreticulin surface exposure (flow cytometry; % calreticulin + cells) and **(F)** HMGB1 secretion (ELISA; pg/mL) at 0, 4, 8, and 12 Gy irradiation in normal vs. M2-conditioned medium, demonstrating dose-dependent ICD signaling attenuated by M2-conditioned medium (∼40% reduction in calreticulin exposure, p < 0.05). Data presented as mean ± SD; n = 3 independent experiments. *p < 0.05, **p < 0.01, ***p < 0.001 (Student’s t-test).

To validate single-cell transcriptomic findings in a functional model, RT-qPCR analysis was performed in HepG2 (ATCC HB-8065) and Huh-7 cells under M2 macrophage-conditioned medium (tumor microenvironment simulation) versus normal medium controls. M2-conditioned cells demonstrated significant upregulation of SPP1 (HepG2: 3.47-fold, Huh-7: 3.29-fold; both p < 0.001), CXCL8 (4.15-fold; 3.88-fold; p < 0.001), APOE (2.82-fold; 2.67-fold; p < 0.01), and CCL3 (2.61-fold; 2.44-fold; p < 0.01) compared to controls. CCL5 and CXCR4 were also significantly upregulated (p < 0.05). ELISA quantification confirmed elevated protein levels of SPP1 (osteopontin), CXCL8 (IL-8), CCL3 (MIP-1α), IL-10, VEGFA, and TGF-β1 in culture supernatants from both conditioned cell lines versus controls (all p < 0.01). These results demonstrate that tumor cells can respond to macrophage-derived immunosuppressive signals by upregulating SPP1, CXCL8, and related mediators at both transcriptomic and protein levels, consistent with the immunosuppressive landscape identified in the scRNA-seq analysis. Radiation dose-response experiments further demonstrated that irradiation (4–12 Gy) induced calreticulin surface exposure, HMGB1 secretion, and ATP release in both HepG2 and Huh-7 cell lines in a dose-dependent manner, confirming radiation-driven ICD signaling in HCC cells. However, co-treatment with M2-conditioned medium attenuated radiation-induced calreticulin exposure by approximately 40% (p < 0.05), suggesting that the immunosuppressive macrophage secretome may partially dampen radiation-induced ICD—providing mechanistic rationale for interventions targeting M2 macrophage-mediated suppression to enhance SBRT immunogenicity. We acknowledge that the conditioned-medium model captures only the paracrine component of M2 macrophage–tumor cell interactions; co-culture systems incorporating THP-1-derived M2 macrophages, primary CD8^+^ T cells, and HCC cells represent a necessary next experimental step. RT-qPCR validation data are presented in Figures 8A,B; ELISA results in Figures 8C,D; radiation ICD data in Figures 8E,F.

## 4 Discussion

This study provides an integrative single-cell transcriptomic characterization of the HCC immune microenvironment across normal liver, primary tumor, and PVTT tissues, validated with an *in vitro* functional model. The central finding—that myeloid dominance and multi-checkpoint T cell exhaustion define a profoundly immunosuppressive TIME in HCC—carries direct translational implications for understanding SBRT-based radio-immunotherapy. These baseline immunosuppressive features constitute structural barriers to radiation-induced anti-tumor immunity and provide mechanistic hypotheses regarding differential radiosensitization that warrant further experimental and translational validation.

The dramatic myeloid expansion in the HCC TIME (reaching 46.5% of tumor cells and 65.0% of PVTT cells) represents a structural barrier to radiation-induced anti-tumor immunity. Tumor-associated macrophages, predominantly M2-polarized in our scRNA-seq dataset, suppress cytotoxic T cell responses through IL-10 and TGF-β1 secretion, upregulate VEGFA to promote immune-excluded vascularity, and maintain T cell exhaustion via SPP1-CD44 and CCL3-CCR5 signaling (Cassetta et al., 2018; Noy and Pollard, 2014). Emerging evidence implicates myeloid cells as dominant determinants of immunotherapy resistance in HCC (Zhu et al., 2014), and our *in vitro* data extend this concept to the SBRT context: M2-conditioned medium drove significant upregulation of SPP1, CXCL8, IL-10, VEGFA, and TGF-β1 in HCC cell lines, and attenuated radiation-induced calreticulin surface exposure by approximately 40%—suggesting that the immunosuppressive macrophage secretome may dampen SBRT-induced ICD signaling, creating a self-reinforcing radioresistant loop that represents a rational therapeutic target.

Emerging evidence further demonstrates that cell death network rewiring—encompassing adaptive transitions between apoptotic, pyroptotic, necroptotic, and ferroptotic programs under immunosuppressive conditions—represents a key mechanism of resistance to combined radio-immunotherapy, whereby the M2-dominant, radioresistant TIME phenotype characterized in this study may reflect a fundamentally altered cell death landscape that blunts the immunogenic consequences of SBRT and limits therapeutic synergy with immune checkpoint blockade (Tang et al., 2019; Guo et al., 2026). Notably, pyroptosis has been shown to drive inflammatory cascades and organ dysfunction in sepsis models (Wang et al., 2025), suggesting that cell death pathway selection critically modulates the immunogenic versus tolerogenic consequences of acute cellular stress in inflammatory microenvironments.

SPP1 emerged as the dominant upregulated immunosuppressive gene in the HCC TIME, consistent with recent landmark studies identifying SPP1+ TAMs as a distinct immunosuppressive macrophage subpopulation promoting angiogenesis, T cell exclusion, and resistance to immune checkpoint blockade (Kuang et al., 2009; Liu et al., 2023). SPP1-expressing TAMs represent a mechanistic link between immunosuppression and vascular invasion—two key drivers of HCC treatment failure—as evidenced by its strong association with PVTT-like myeloid dominance in our scRNA-seq data. The CXCL8-CXCR1/2 axis (log2FC = 3.14) promotes myeloid recruitment and creates a self-reinforcing immunosuppressive loop; this may be particularly problematic in SBRT settings where radiation-induced TNF-α could further drive CXCL8 production, paradoxically recruiting immunosuppressive myeloid cells to the irradiated field (Barker et al., 2015). Inflammatory cytokine modulation—including attenuation of circulating cytokine axes through lipid-modifying interventions—has demonstrated mechanistic feasibility in systemic inflammatory states (Li et al., 2025), supporting the rationale for targeting the CXCL8-driven myeloid recruitment loop as an adjunct to SBRT in HCC.

Multi-checkpoint T cell exhaustion—characterized by co-expression of PDCD1, LAG3, TIGIT, and CTLA4 in 75.7% of tumor-infiltrating T cells—represents the adaptive arm of immune evasion and a critical barrier to radiation-induced T cell reactivation. The presence of multi-checkpoint exhaustion indicates that single-agent anti-PD-1 therapy is likely insufficient to restore functional anti-tumor immunity in these patients. Recent clinical data support dual checkpoint strategies in HCC: the combination of nivolumab and ipilimumab achieved 31% ORR in pretreated HCC (Yau et al., 2020), and the HIMALAYA trial demonstrated superiority of tremelimumab plus durvalumab over sorafenib (Abou-Alfa et al., 2022). Our single-cell data identifying LAG3 and TIGIT as dominant co-inhibitory receptors alongside PD-1 provide mechanistic rationale for incorporating LAG3 or TIGIT blockade with SBRT, as radiation-induced antigen release may potentiate T cell reactivation when multiple exhaustion checkpoints are simultaneously blocked (Yost et al., 2019).

Mechanistically, the intersection of programmed cell death pathways with immune checkpoint regulation in the tumor microenvironment has emerged as a key determinant of immunotherapy response and resistance: distinct cell death modalities differentially regulate co-inhibitory ligand expression, antigen cross-presentation efficiency, and dendritic cell activation capacity, providing a molecular rationale for combining cell death-inducing modalities such as SBRT with multi-checkpoint blockade in HCC (Galluzzi et al., 2017; Zhang et al., 2025).

The myeloid-endothelial cell interaction network—dominated by VEGFA signaling—reveals an immunovascular co-regulatory axis particularly amenable to combined anti-angiogenic and radiation intervention. VEGFA-driven angiogenesis creates an immune-excluded tumor architecture by establishing high-pressure, poorly perfused vessels that physically impede immune cell infiltration and may reduce intratumoral oxygen tension, limiting radiation efficacy (Fukumura et al., 2018). Normalization of tumor vasculature by anti-VEGF agents can paradoxically enhance immune infiltration and improve radiation dose distribution within the tumor—providing mechanistic synergy between anti-angiogenics, immunotherapy, and radiotherapy. The strong VEGFA expression in our scRNA-seq data (log2FC = 2.03 in tumor vs. normal) and its upregulation in M2-conditioned HCC cells support further investigation of anti-VEGF/SBRT/ICI combinations, particularly in preclinical models of PVTT-like myeloid dominance (Kudo, 2020).

This study has important implications for the design of novel radiotherapy combinations targeting the HCC TIME. First, macrophage reprogramming strategies—including CSF1R inhibitors, TLR agonists (e.g., poly-ICLC), or CD47 blockade—may convert M2-dominant TAMs toward M1 phenotype, disrupting the myeloid immunosuppressive niche and potentially restoring radiation-induced ICD capacity (Mantovani et al., 2017). Second, SPP1-targeted interventions may simultaneously disrupt macrophage-mediated immunosuppression and PVTT-promoting signaling. Third, FLASH radiotherapy—which delivers ultra-high dose rates preferentially sparing normal tissues while maintaining equivalent tumor control—may be particularly advantageous in the myeloid-dominated HCC TIME by potentially inducing tumor-specific ICD while limiting the irradiation-induced pro-inflammatory cytokine release that could amplify myeloid recruitment (Bourhis et al., 2019). Fourth, combining SBRT with LAG3/TIGIT blockade and CSF1R inhibition represents a theoretically optimal triple-combination approach to simultaneously overcome the two dominant immunosuppressive barriers identified in this study (myeloid dominance and multi-checkpoint exhaustion), though this hypothesis requires prospective validation.

Several limitations of this study must be acknowledged. The scRNA-seq characterization is anchored to GSE149614 (single patient, HCC07, stage IIIB); patient-level heterogeneity in TIME composition remains an important source of variability requiring prospective multi-center scRNA-seq studies with cross-dataset integration. The *in vitro* validation employs a conditioned-medium model that captures only the paracrine effect of M2 macrophage secretome on HCC cells, and cannot directly recapitulate myeloid-T cell interactions that are central to the scRNA-seq-derived findings. For DEG analysis, Benjamini–Hochberg FDR correction was applied genome-wide (FDR <0.05); for GSEA, adjusted p-values (FDR q < 0.25, NES >1.5) are reported per established convention. For *in vitro* RT-qPCR and ELISA experiments (n = 3 independent replicates), the sample size limits statistical power and these results should be considered hypothesis-generating. The cross-sectional scRNA-seq design precludes characterization of how SBRT itself remodels the TIME over time; future longitudinal *in vivo* or *ex vivo* models are required to evaluate radiation-induced TIME remodeling. Future studies should incorporate: (1) multi-patient scRNA-seq datasets with matched transcriptomic annotations to address patient-level heterogeneity; (2) co-culture systems incorporating THP-1-derived M2 macrophages, primary CD8^+^ T cells, and HCC cells; and (3) functional *in vivo* validation in syngeneic HCC mouse models.

### 4.1 Limitations

Key limitations include: (1) the scRNA-seq analysis is based on GSE149614 (single patient, HCC07, stage IIIB), which limits generalizability; prospective multi-patient multi-center cohorts are required to fully resolve patient-level heterogeneity; (2) the conditioned-medium model approximates only paracrine M2-tumor cell interactions—direct myeloid-T cell-tumor co-culture systems are required; (3) the radiation ICD experiments use single-fraction irradiation in cell lines, which does not replicate multi-fraction SBRT dosing schedules; (4) *in vitro* experiments (n = 3) have limited statistical power and are hypothesis-generating; and (5) the cross-sectional design cannot characterize dynamic TIME changes induced by SBRT *in vivo*. Functional validation in syngeneic *in vivo* HCC models is required.

## 5 Conclusion

This study provides an integrative single-cell transcriptomic characterization of the HCC immune microenvironment across normal liver, primary tumor, and PVTT tissues, anchored by single-patient scRNA-seq analysis (GSE149614; HCC07, stage IIIB) and validated by *in vitro* RT-qPCR, ELISA, and radiation dose-response experiments in ATCC cell lines. Our findings demonstrate that myeloid-dominated immunosuppression and multi-checkpoint T cell exhaustion define a profoundly immunosuppressive TIME in HCC. Key immunosuppressive mediators including SPP1, CXCL8, TGF-β1, and VEGFA are validated at transcriptomic and protein levels. Critically, the M2 macrophage secretome attenuates radiation-induced ICD signaling in HCC cells by ∼40%, providing direct *in vitro* evidence that the immunosuppressive TIME may dampen SBRT immunogenicity. These data provide a mechanistic framework for rational combination of SBRT with immune reprogramming strategies—including macrophage reprogramming, dual checkpoint blockade, and anti-angiogenic agents—to overcome the immunosuppressive HCC TIME and maximize durable tumor control. Future mechanistic and *in vivo* studies are required to validate these findings and identify candidate biomarkers for SBRT-based radio-immunotherapy strategies.

## Data Availability

The single-cell RNA-seq data analyzed in this study are publicly available in GEO under accession GSE149614 (https://www.ncbi.nlm.nih.gov/geo/query/acc.cgi?acc=GSE149614). Additional analysis resources are included in the article/Supplementary Material. Further inquiries can be directed to the corresponding authors.
